# Determining Priorities in the Aboriginal and Islander Mental Health Initiative for Youth App Second Phase Participatory Design Project: Qualitative Study and Narrative Literature Review

**DOI:** 10.2196/28342

**Published:** 2022-02-18

**Authors:** Josie Povey, Michelle Sweet, Tricia Nagel, Anne Lowell, Fiona Shand, Jahdai Vigona, Kylie M Dingwall

**Affiliations:** 1 Menzies School of Health Research Charles Darwin University Casuarina Australia; 2 Menzies School of Health Research Charles Darwin University Adelaide Australia; 3 Northern Institute Charles Darwin University Darwin Australia; 4 Black Dog Institute, University of New South Wales Sydney Australia; 5 Menzies School of Health Research Charles Darwin University Alice Springs Australia

**Keywords:** Aboriginal and Torres Strait Islander, young people, digital mental health, app, participatory design, decision-making, mobile phone

## Abstract

**Background:**

Digital mental health tools can promote access to culturally safe early intervention mental health services for Aboriginal and Torres Strait Islander young people. Participatory design methodology facilitates user engagement in the co-design of digital resources. However, several challenges have been identified that limit the methodological rigor of this approach.

**Objective:**

This paper aims to present an in-depth account of the second phase of participatory design in the development of the Aboriginal and Islander Mental Health Initiative for Youth (AIMhi-Y) app.

**Methods:**

A *first idea* storyboard, generated from a formative phase of the AIMhi-Y project, was refined through a series of youth co-design workshops and meetings. A narrative review of the literature, 6 service provider interviews, and engagement with an expert reference group also informed the design process. Generative design activities, storyboarding, discussions, and voting strategies were used.

**Results:**

The participatory design process identified the app features preferred by young people and service providers and assessed their alignment with current recommendations from the scientific literature. Findings from the co-design process are presented across 9 app characteristic domains. Integration of findings into app design proved complex. Although most preferred features identified by young people were included to some degree, other inclusions were restricted by budget, time, and the need to integrate best practice recommendations. A process of prioritization was required.

**Conclusions:**

Participatory design is often cited in the development of digital mental health resources; however, methods are diverse and often lack detailed descriptions. This study reports the outcomes and strategies used to determine priorities in the second phase of the development of the AIMhi-Y app. We provide an example and the key learnings to inform others seeking to use participatory design with a similar cohort.

## Introduction

### Background

Aboriginal and Torres Strait Islander young people possess many strengths that stem from an ancient and resilient culture [1]. However, they also experience negative influences on well-being, mental illness, and suicide at higher rates than their non-Indigenous counterparts [2,3]. Help-seeking is impeded by stigma, shame, differences in language and culture, geographical isolation, and cost [4-7]. Mental health services that integrate Aboriginal and Torres Strait Islander people’s holistic health worldviews are recommended [8] but remain limited [4]. Culturally responsive technological innovations offer the potential to bridge mental health service gaps and address increasing demand [9-12].

Digital mental health (dMH) resources include “mental health, suicide prevention, and alcohol and other drugs services delivered via a digital platform” [13]. These can be stand-alone, clinician-supported, or used alongside face-to-face services [13]. Evidence supporting the acceptability and effectiveness of dMH solutions for Aboriginal and Torres Strait Islander young people is emerging [9,14-17]. Nevertheless, culturally adapted dMH resources remain limited [18,19]. End user involvement in the design of dMH interventions is recommended [20,21] and assists in valuing Aboriginal and Torres Strait Islander expertise [11] and decolonizing research processes [22,23].

Participatory design is often used in dMH development to engage users. It is a multistage process (defining the problem, identifying solutions, and generating and evaluating prototypes), with end user involvement throughout [21,24,25]. Research tools can include brainstorming, storyboarding, scenarios, prototyping, role plays, 2D sketching, group discussion, and user journeys [24,26]. Participatory design upskills and engages end users in experiential action-based activities with shared informed decision-making throughout [21,27]. Integration of best practice evidence and expert advice [21,26], visibility of outcomes [28,29], adequate time and resourcing [30], and a flexible iterative approach [30] are recommended to ensure success.

Although considered best practice, several challenges exist in undertaking participatory design. Tensions in preferences among designers; end users; and therapy and pedagogy experts, whose input is vital in designing resources that are appealing, usable, and address therapeutic goals, can prove challenging [31,32]. In addition, differences in user preferences based on age, gender, video game familiarity, and mental health need often arise [19,33-35]. Strategies to address these predictable tensions between and within stakeholder groups and manage selection bias include the following: defining roles, upskilling participants, developing a positive team approach, and supporting meaningful engagement [30,36,37]. Such strategies value end user input and help overcome power differentials [19,33-35,38]. In addition, co-design in cross-cultural contexts requires specific engagement strategies that embrace language and worldview differences and empower Indigenous young people throughout [39,40]. In summary, successful participatory design characteristics include the following:

- End user participation throughout
- Finding balance between tailoring-to-context and a one-size-fits-all approach
- Generation of resources through experiential and playful action-based activities
- Shared decision-making throughout, which is transparent and iterative
- Ensuring the empowerment, upskilling, and safety of participants
- Strategies to address predictable tensions between user preferences and other stakeholder groups (eg, app developers, researchers, clinicians, and educators)
- Strategies to address differences in end user preferences related to gender, age, mental health need, and technological familiarity
- Strategies to balance stakeholder expectations and resource availability (eg, time and budget)

Although recommended [24,26], in-depth reporting of processes used throughout the participatory design of dMH tools remains scarce [41], with some notable exceptions [36,42]. This omission inhibits the development of participatory design as a sound methodological approach and limits the understanding of *how* and *if* participatory design increases effectiveness, reach, adoption, and implementation of dMH resources [33,43,44].

The Aboriginal and Islander Mental health initiative (AIMhi) program of research develops, tests, and implements dMH resources for Aboriginal and Torres Strait Islander people [45,46]. Resources such as the Stay Strong Plan, a culturally adapted low-intensity cognitive behavioral therapy (CBT) intervention (available in digital and paper-based formats) have demonstrated effectiveness in reducing psychological distress and substance misuse among adults [16,47]. Developed and implemented over a decade within the Greater Darwin and Tiwi Island regions, the initiative is recognized and trusted within the community. Community feedback, suggesting changes to appeal to young people [9,10], led to the commencement of a youth-focused program of research (Aboriginal and Islander Mental Health Initiative for Youth [AIMhi-Y]). Phase 1, reported elsewhere [48], aimed to understand Aboriginal and Torres Strait Islander young people’s lived experience of mental health and well-being, views on dMH resources, and design and content preferences. Preferred features included a smartphone-based app that incorporates strengths-based mental health information presented through relatable storytelling and a fun, appealing, easy-to-use interface, which encouraged app progression [48]. Phase 1 resulted in the first 2D storyboard, which was used as a basis for this second design phase.

### Objective

Using a participatory design approach, this study (phase 2) aims to iteratively enhance the AIMhi-Y app storyboard and finalize the first release of the AIMhi-Y app. This was done through collaboration with Aboriginal and Torres Strait Islander young people, service providers, integration of evidence and best practice recommendations, and iterative usability testing with the app developer. In this paper, we provide an in-depth report of our findings and experiences in developing the AIMhi-Y app.

## Methods

### Study Design

**Figure 1:**
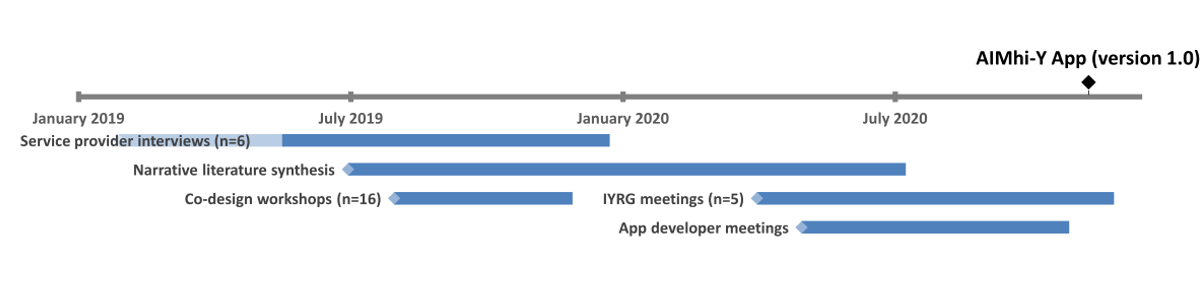
Timeline of phase 2 Aboriginal and Islander Mental Health Initiative for Youth (AIMhi-Y) app development project activities. IYRG: Indigenous youth reference group.

This 2-year qualitative participatory design project conducted youth co-design activities (workshops and meetings), service provider interviews, a narrative synthesis of the literature, and app developer meetings. Several activities occurred concurrently as shown in Figure 1.

### Participant Selection and Setting

Purposive sampling was used to recruit young people and service providers with experience in working with young people across 3 sites (2 schools and 1 residential drug rehabilitation facility). All 3 sites that engaged in phase 1 [48] continued their engagement in phase 2. Youth participants invited to the co-design workshops were nominated by the teaching or support staff at their site. Inclusion criteria were as follows: Aboriginal or Torres Strait Islander descent, aged 8-18 years, and willing to talk in a group setting in English (at urban and remote sites) or Tiwi language (at the remote site). Service providers were key contact persons at each site and their engagement with the project ranged from 6 months to 2 years.

After completing the co-design workshops at the school and residential rehabilitation facility, a separate group of young people was recruited to form an Indigenous youth reference group (IYRG). This smaller group provided prompt feedback throughout the app development stage. Recruitment occurred via email and social media advertising, disseminated through project networks. Individuals identifying as Aboriginal or Torres Strait Islander, aged 15-25 years, and willing to attend meetings via videoconference (owing to the COVID-19 social distancing regulations) were recruited to the IYRG. Participants involved in the phase 1 or phase 2 co-design workshops were prioritized throughout recruitment, aiming for diverse age and gender representation.

Informed oral consent was obtained (face-to-face or via phone) from all participants and from guardians of those aged <16 years before participation. A pictorial consent flip chart and participant information handouts were used to assist communication. Interpreters were available for those who spoke a language other than English at home. The first author (JP) led the project, supervised by 5 senior researchers with clinical and dMH expertise. In addition, guidance from 4 Indigenous researchers (n=2, 50% youth and n=2, 50% women) played a vital role in establishing rapport, developing and refining data collection activities, and interpreting during face-to-face discussions. Research team members were trained in distress management and suicide awareness. Site-specific risk management processes were agreed upon by each organization before commencement. They included the provision of immediate safety measures by researchers, notification of senior research team members, and follow-up and referral to treatment services provided by the nominated site contact person.

An existing expert reference group (established in phase 1) included 12 service providers and researchers with relevant expertise from mental health, drug and alcohol, child protection, and education sectors (n=3, 25% men and n=5, 42% Aboriginal). The expert reference group met or were contacted via email biyearly to advise on research processes, app design, and content [48]. 

### Ethical Approval

Ethical approval was obtained from Menzies School of Health Research Human Research Ethics Committee (HREC 2017-2991), including the Indigenous subcommittee and the Northern Territory Department of Education Research Ethics Committee (reference number: 13417).

### Data Collection

#### Co-design Workshops and IYRG Meetings

A total of 16 co-design workshops and 5 IYRG meetings were held with youth participants. Workshops and meetings used generative and discussion methods to design and refine app attributes [24]. Participants were presented with previous findings, mock-ups, storyboards, and app prototypes. Voting (show of hands) and discussion strategies were used to reach a consensus. Co-design workshop and meeting activities (Textboxes 1 and 2) were developed, informed by the literature and research team consultation.

**Co-design workshop activities guide.**

**Workshop 1**

**Figure 2:**
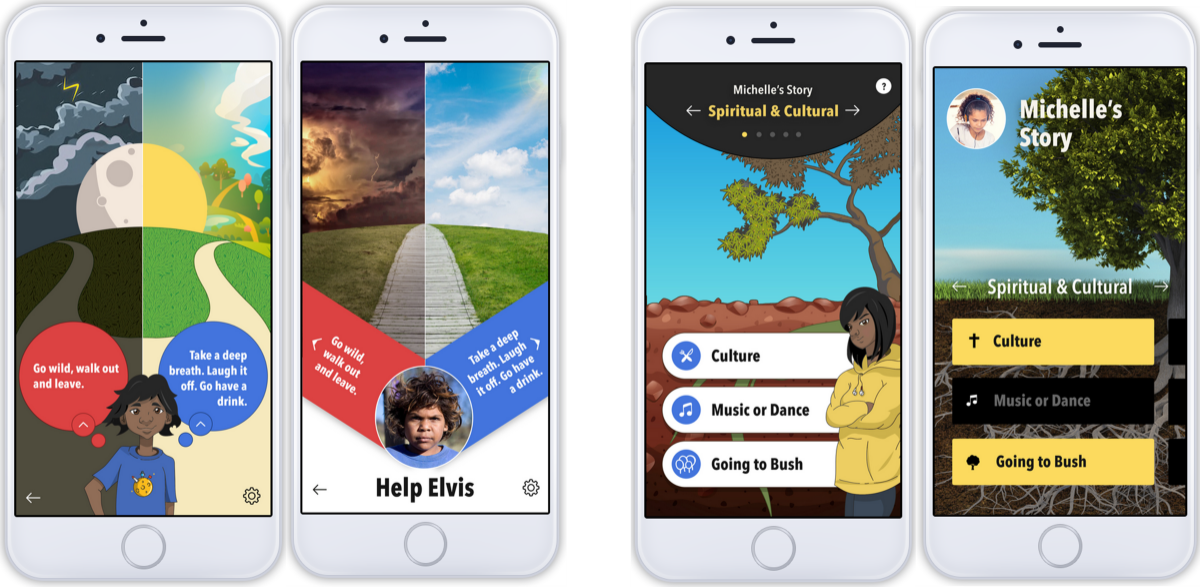
Prototypes used to elicit discussion on design attributes.

- User flow diagrams (paper-based 2D visual representations; A0 size) integrating the app storyboard drafted in phase 1; oriented and educated young people to purpose, features, and content through a minilecture.
- Interactive group discussion; elicited participants’ preferred features, interactive components, and usability preferences.
- Introduction of mock-ups (Figure 2) using printed worksheets; initiated group discussion and written feedback on design styles, storylines, and character attributes.

**Workshop 2**

- Indigenous researcher-drawn home page mock-ups; initiated group discussions about metaphors, reward systems, and progress displays.
- Young people generated their own home page ideas through individual and small group sketch activities.
- Revised design styles (based on workshop 1) were introduced and discussed. Voting (show of hands) was conducted to reach an agreement on look and feel.

**Workshop 3**

- Video scripts based on lived experience examples provided from phase 1 [48] were developed by the research team and presented to the group through interactive discussion. Young people were asked to draw a series of scenes to complete the short video. Sketching and discussion explored participant’s understanding of information, preferred perspectives (first person, third person, or noncharacter delivered), imagery, storylines, character strengths, challenges, and personal attributes.

**Workshop 4**

- A total of 3 youth-generated ideas for app narratives and metaphors (generated within workshop 2) were presented. Young people sketched and described a mock-up of their preferred narrative or generated a new one.

**Indigenous youth reference group meetings activities guide.**

**Meeting 1**

- Group members were presented with relevant findings from phase 1 and the co-design workshops through a minilecture. A revised app storyboard oriented the participants to overall aim, app narrative, structure, and characters.
- Indigenous youth researcher–generated logo mock-ups prompted group discussion.
- Visual aids prompted discussion of preferred reward systems, background images, color schemes, and design element preferences.

**Meeting 2**

- Iterated versions of the logo, backgrounds, and reward images were presented for discussion.
- Researcher-generated digital home page mock-ups prompted group discussion.
- Persona development activities prompted group discussion and explored the character’s attributes such as family, strengths, cultural identity, and language.

**Meeting 3**

- Completed backgrounds and logo were presented for confirmation by group members.
- Storyboard activities and group discussion completed a detailed review of narrative, wording, reward metaphors, and images for Ramone quest.
- Character names and support person roles were reviewed and discussed.

**Meeting 4**

- Draft app prototype was presented using screen-sharing software. Initial impressions and feedback were elicited through group discussion.
- A researcher-generated mock-up of rewards page prompted group discussion.

**Meeting 5**

- Aboriginal and Islander Mental Health Initiative for Youth app prototype 1.0 presented with feedback elicited for integration at a later stage.
- Pilot study procedures were presented and discussed. Strategies for recruitment and consent were discussed, with changes made in line with recommendations from participants.

Workshops were facilitated by the first author (JP) and 2 youth Indigenous researchers (JV), promoting group rapport and enabling an insider perspective [49]. A senior Indigenous researcher, who was an interpreter and community elder, was present at all workshops in the remote school, offering participants the option to engage using their first language. Several unsuccessful attempts to involve an interpreter at the residential rehabilitation center were made. This was considered important as few of these participants spoke English as their first language, in contrast to the youth participants from the urban schools. The research team acknowledges that the presence of interpreters would have likely deepened discussion and improved rapport [6]. Workshops began with an icebreaker activity, introductions, sharing of food, and discussing kinship and family relationships. Workshops were 40 to 90 minutes in duration. Groups were divided by gender to follow local cultural protocols [50]. Young people who identified as gender diverse chose the group with which they felt most comfortable to work. Larger groups (>10 people) were divided into self-selected peer groups of 3 to 5 people. Groups rotated around activities in a classroom environment. Workshops were audio recorded and complemented by field notes.

The IYRG meetings were facilitated by the first author (JP), a youth Indigenous researcher (JV), and a senior researcher (MS) and held via videoconference after school. Meetings started with introductions, icebreaker activities, and discussing kinship and family relationships. Meetings were 90 minutes in duration and members were reimbursed for their time (Aus $75 [US $54] sitting fee).

#### Service Provider Interviews

A total of 6 semistructured in-depth interviews of 40 to 60 minutes duration with service providers were conducted concurrently with youth co-design activities. Interviews aimed to gain feedback on the app storyboard, refine features, and explore implementation considerations. Interviews were conducted at the study site or over the phone by the first author (JP), with an Indigenous researcher (JV) present on 2 occasions. Service provider interview guides (Multimedia Appendix 1) were informed by the literature and refined within the research team.

#### Narrative Literature Synthesis

A narrative literature synthesis was undertaken to inform app development. Searches used a combination of search terms, including mental health, technology use, adolescence, Indigenous populations, therapeutic interventions, and efficacy. Inclusion criteria were broad, aiming to capture evidence for dMH interventions across multiple population groups (youth, adult, and Indigenous). Exclusion criteria included articles not published in English owing to limited resources. Searches were conducted in multiple web-based databases, including EBSCOhost, Scopus, CINAHL, PubMed, Google Scholar, Informit, and organization websites (Multimedia Appendix 2) and included literature published from 2000-2020. Several dMH development guidelines and safety standards were included in the review. Multiple searches were conducted between July 2019 and July 2020, and the findings were used to inform the design process. Therefore, the specific number of articles found and excluded was not recorded. The first author (JP) conducted these searches and formulated the synthesis of findings from the review of relevant literature. Other authors (KMD and TN) provided feedback and guidance for the identification of additional relevant literature. References were uploaded to the Endnote referencing system (Clarivate) and categorized into groups.

### Analysis

The first author (JP) transcribed all the sections of the co-design workshop recordings that were relevant to the design process. The first and second authors independently thematically coded data from workshops, meetings, and interviews by combining an inductive process, generating codes from the data (MS) [51], with deductive analysis, based on the app preferences identified in phase 1 (JP) [52]. Following consensus, further discussions were conducted with the full research team. Scientific literature was concurrently synthesized into table format by the first author (JP) and thematically analyzed according to the recommended dMH attributes. The themes emerging from youth co-design workshops, meetings, and service provider interviews were then integrated into the themes identified through the literature review. Findings from all analyses informed the design and development of the AIMhi-Y app (version 1.0). The dMH safety standards and youth preferences were prioritized throughout development. Analysis occurred before and concurrent to meetings between the app developer and the research team to translate design preferences into prototypes. Demographic data were analyzed for descriptive statistics to describe the sample.

## Results

### Participant Demographics

#### Co-design Workshops and Meetings

A total of 16 co-design workshops (5 groups with a series of 2-4 workshops) and 5 IYRG meetings involved 75 young people (Table 1). Participants were primarily female (40/75, 53%) and currently engaged in school (66/75, 89%; Table 2). A total of 10 young people (n=6, 60% remote and n=4, 40% urban) continued engagement from phase 1 [48]. In all, 38% (6/16) IYRG participants were involved in previous co-design workshops. Researcher observations of participant body language and interaction within the group indicated that engagement in workshop activities ranged from disinterest to enthusiastic participation in consultation and co-design and was influenced by multiple factors, including participant mood, research activity, and facilitator. Flexibility in the workshop design allowed participants to engage and reengage at will. On one occasion, a young person expressed distress unrelated to workshop content and was provided immediate support by the research team, with follow-up by the identified site contact person, as per the agreed risk management processes.

**Table 1:** Number of youth participants at each co-design workshop or meeting (N=75).

Workshop or meeting	Remote School, n (%)		Urban School, n (%)		Rehabilitation service, n (%)	IYRG^a^, n (%)
	Male	Female	Male	Female	Mixed gender	Mixed gender
1	7 (9)	6 (8)	12 (16)	19 (25)	4 (5)	16 (21)
2	6 (8)	5 (7)	11 (15)	15 (20)	5 (7)	14 (19)
3	7 (9)	6 (8)	8 (11)	16 (21)	N/A^b^	8 (11)
4	9 (12)	7 (9)	N/A	N/A	N/A	6 (8)
5	N/A	N/A	N/A	N/A	N/A	5 (7)

^a^IYRG: Indigenous youth reference group.

^b^N/A: not applicable.

**Table 2:** Co-design workshop and Indigenous youth reference group participant demographics.

Variable			Values
**Gender (N=75), n (%)**			
	Female	40 (53)	
	Male	33 (44)	
	Gender diverse	2 (3)	
Age (years; n=56^a^), mean (SD; range)			15.14 (1.74; 8-18)
Reside in a very remote community (n=65^a^), n (%)			24 (37)
English not the main language spoken at home (n=56^a^), n (%)			21 (38)
Not currently engaged in school (n=65^a^), n (%)			7 (11)
Owned a smartphone (n=56^a^), n (%)			41 (73)

^a^Owing to missing data.

#### Service Provider Interviews

A total of 6 service provider interviews (4, 67% men and 2, 33% Aboriginal) across 3 sites included teachers (2/6, 33%), school or service support personnel (3/6, 50%), and a manager (1/6, 17%).

### Youth, IYRG, and Service Provider App Preferences

#### Overview

Most youth participants held strong preferences for activity types, engagement strategies, interface, graphic design, and language preferences. Safety, security, psychological approach, evidence, implementation, and accessibility features, although valued by some young people, were more prominent throughout service provider preferences. We present the findings from co-design activities in the following sections.

#### Activity Types

Young people’s preferred activity types included *videos, minigames, and self-monitoring* of well-being and goals*.* Activities that incorporate *strengths-based mental health information* and *skill development* were preferred:

...It’s looking like a good app...Add more videos...[make them] short, talking about mental health and wellbeing...[I] like the depth of the animal game with the mindfulness part...

Female participants, aged 15-17 years, IYRG members

Activity types revealed many diverse opinions from youth and service providers. Participants suggested that drivers of differences in preferences were related to age, gender, video game familiarity, community of origin, school attendance, level of distress, and cultural connectedness:

...if it's like a really young one, they will be looking onto the [mini] game, then they feel more comfortable...if a female client, [who is] really worried; really down; very lonely...she may be looking onto something else...kids who're having significant absence from school, they don't [respond to trophies or points]...[they] need a reward in that they're getting more - more games, more activities...

Service provider

#### Engagement

Participants valued options to *customize, personalize, represent progress, gamify, prompt real-world action, allow exploration,* and *provide unlimited use*:

...is this one of the characters you get to customize...choose what they are wearing?

Female participant, aged 13 years, co-design workshop

...as you went down the path...have footsteps to show you have moved...the sound of feet crunching on dirt...things that...reflect the decision you made...

Female participant, aged 15 years, co-design workshop

...[include] motivational things that make you get up and do something...like star jumps...to get you moving...or like 'judging on the questions you answered today...it would be helpful for you to do...'

Female participant, aged 15 years, co-design workshop

...if they're really enjoying it, and they finish it...Is there something that they can keep on going back to? Like their goals – that keep changing or updating?

Service provider

Participants valued *storytelling* with relatable characters, which helped build trust and prompt reflection. Stories told from different perspectives, including peers and family members, were necessary. The character’s gender influenced some young people’s willingness to listen:

[I] like that he is telling his own story...because that would help other people overcome what they are going through as well...

Female participant, aged 16 years, co-design workshop

Male or female I would listen because that...is their experience in life...I reckon girls are more interesting than boys...we already know everything about boys, but understanding girls would be better...

Male participant, aged 16 years, co-design workshop

Nah, I wouldn't listen to it as much [if the character was female]...[Include] two different stories - for girl - like teasing and gossip and anxiety - or some thing more serious like for boys...

Male participant, aged 15 years, co-design workshop

**Figure 3:**
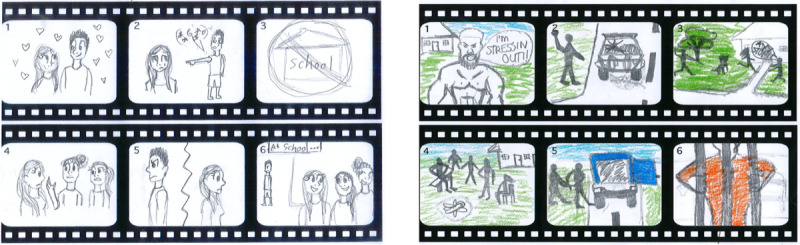
Participant-drawn storyboards: 'Relationship problems' and 'body stressing'.

Figure 3 provides selected youth-drawn storyboards and descriptions highlighting story content and imagery suggestions.

The description of the storyboard, *body stressing*, was as follows:

[He is] feeling stressed can lead to sickness, drugs and alcohol and car crash, [he is an] angry man...getting in trouble with police – gettin' sent to prison...he is trying to kill himself...he gotta stop gunja (marijuana), be with family...recovery...he [character] should tell the story - he should tell it after [it's happened]...Mum as well - yeah she can tell her part of the story...[and] uncle for taking him out hunting, like see the difference.

Male participant, aged 18 years, co-design workshop

The description of the storyboard, *relationship problems*, was as follows:

...there is a girl, she has worries with [her] boyfriend and gossip...not going to school, fighting with her mum, focusing on her boyfriend too much and texting all the time, staying up late, or fake going to sleep early...[she is feeling] sad, depressed. He is stressing her out, he want money, she is jealous...he is jealous of her, he wants to fight, about another boy, he is really clingy...[Then] she dumps the boyfriend and has her own life...talks to her best friend...she helps her out to dump the boy, she takes her back to school, forget about that boy, she makes different excuses to get away from him...she talks to grandmother and grandmother talks to boyfriends parents and says keep away from my grandaughter.

Female participant, aged 18 years, co-design workshop

#### Interface and Graphic Design

Participants valued *aesthetic, relevant,* and *intuitive designs,* with diverse opinions on styles and graphics noted:

...some people might like to see like a groovy animation and some might like a more real one...everyone is different.

Female participant, aged 15 years, co-design workshop

...it was [interesting] at first and then it just started drag on a bit...if I was a fisherman I would find it interesting but I don't fish [we] are from [Central Australia]...make it look like it is in the outback, like in community...

Male participant, aged 16 years, co-design workshop

IYRG members often referred to valuing and including diversity of all those involved and incorporated this into their app content and design suggestions:

I like how the desert and bush intertwine. We don't all face same situation [but] we're [Indigenous youth] still interconnected. The trouble that we face is similar no matter where we are.

Female participant, aged 16 years, IYRG Member

Get [images] from all over NT and put in the shapes – incorporate salt water and desert...[include] stories from different places incorporating the [Aboriginal] languages from that place...We live in open places both Top End and Central Desert – needs opening perspective, looking up and out e.g. sunset...

Multiple IYRG members

#### Language

Youth and service providers identified preferences for *audio options* and *integration of Indigenous Languages*:

Is it something you only read, or is there someone going to be reading it?

Male participant, aged 17 years, co-design workshop

Youth participants highlighted preferences for gender-neutral *familiar metaphors and imagery* relating to nature, a journey, making or nurturing something, sport, ceremony, and lore:

Make it look more alive with bird life and plants.

Female participant, aged 16 years, co-design workshop

Going out bush, fishing...driving...over time you can get a campsite, fishing rod...rewards like that...could you like, pick the upgrade...[a] hot chocolate...

Female participant, aged 15 years, co-design workshop

Rewards need to be gender neutral.

Female participant, aged 17 years, IYRG

**Figure 4:**
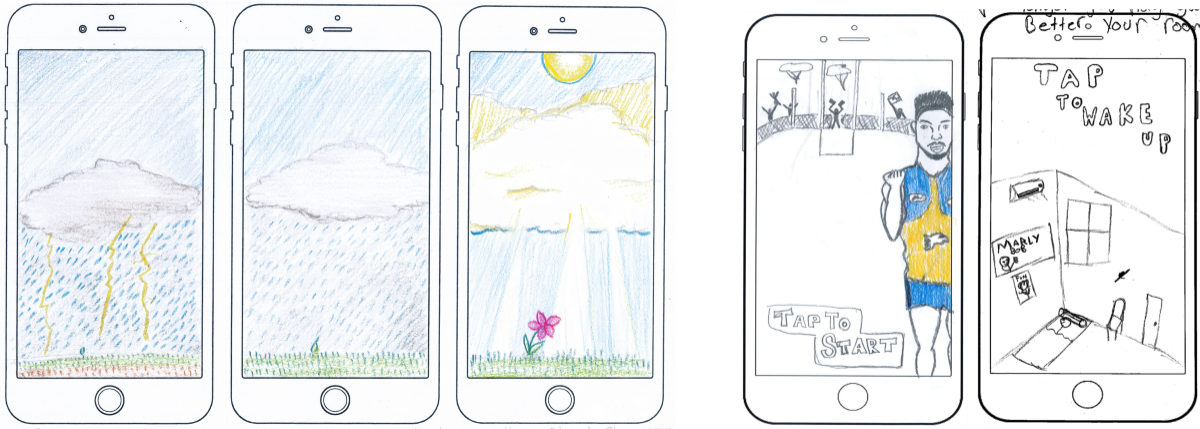
Participants proposed metaphors: 'We all have feelings' and 'Homepage ideas'.

Figure 4 provides youth-drawn images and descriptions of proposed metaphors.

The description of the proposed metaphor, *we all have feelings*, was as follows:

The rainy, stormy, cranky cloud is angry and it wants to burn everything with lightening. But then it realises that it shouldn't be destroying nature, but helping nature grow. The grumpy cloud calms down and stops striking lightening because it can see a little seed trying to grow. The cloud pours rain on the sapling. The cloud stops raining and moves onto the side so the sun could shine on the little sapling. The sapling grows into a beautiful flower. The rain bought nature to life.

Female participant, aged 14 years, co-design workshop

The descriptions of the proposed metaphors, *home page ideas*, were as follows:

Tap game to start...kick the footy to score.

Male participant, aged 18 years, co-design workshop

The longer you play the game the better your room looks, like upgrades...aircon...music...

Male participant, aged 17 years, co-design workshop

#### Safety

Young people and service providers highlighted the importance of *crisis support features*:

I liked on the get help page, you took in consideration different ways people get help...text, phone...

Female participant, aged 17 years, IYRG Member

#### Psychological Approach

Young people and service providers valued strengths-based brief interventions, identifying prototypes that integrated a culturally responsive low-intensity CBT intervention as appealing [48]:

Sometimes we're using that [Stay Strong app] here in our program...You know the yellow colour (representing worries), the green colour (representing strengths) – when it comes, then they [clients] can feel it...if we can consistently use the same thing, they feel like, “Okay. This is the one. We know how to do that...”

Service provider

#### Security, Privacy, and Reliability

Most young people and service providers preferred security features to be optional:

...Do they really know which password they put on last month?...It's not necessary...[But] if they want to, then yeah [include the option].

Service provider

#### Evidence and Implementation

The potential to promote *early intervention* and *continuity of care* was also highlighted by service providers as important; however, it required consideration about the transfer of information among devices:

...We speak to those boys about...going to the school counsellor, and they just don't want to be there...this would be better for us to say, “Have a look at this app”...So, that preventative side of things would be really good.

Service provider

We are trying to connect these people back to the community. So, if...we can just give them [other service providers] – this is what this client was working on...[if] they can use the same password and they already went half-way and they can continue.

Service provider

#### Accessibility

All participants highlighted *free availability on a smartphone* to allow real-time anonymous use*,* with features that accommodate *data use concerns* and *platform compatibility* as preferred:

That's what I like...a lot of them are ashamed but if they're using this [app], it's discreet...no one else knows they are using it...They've all got their phones, it kind of gives them that power...a sense of independence...Is it for free?

Service provider

Service providers also noted that *availability on other devices* was necessary for some settings:

If we can get the app on an iPad?...no one can use phones at school.

Service provider

### Narrative Literature Synthesis

#### Overview

Few papers specific to Indigenous youth populations and dMH apps were identified, suggesting that the field is still emerging. Publications relevant to this synthesis included certification guidelines [13]; evaluative tools [31,53,54]; and systematic reviews relating to Indigenous psychotherapy [55-57], dMH approaches for adolescents [58-62], digital health solutions for Indigenous people [11,63-66], and app features [20,44,64,67,68]. Information about Aboriginal and Torres Strait Islander people’s technology use [12,69] was also included. A total of 3 mental health treatment apps designed to meet the needs of Aboriginal and Torres Strait Islander people were identified, with 1 (33%) app specifically designed for youth [10,15,16,70,71]. In the following section, we present recommendations from the literature across multiple population groups, including youth, adults, and Indigenous populations, recognizing the varied depth of understanding in each field. Multimedia Appendix 3 summarizes recommendations for the development of dMH tools for Indigenous young people [6,9,11-14,16, 17,18,19,31,44,46-48,53-55,57-60,62,63,65,66,68,69,71-100].

#### Therapeutic Approach

Current guidelines recommend that mental health apps incorporate effective therapeutic approaches such as CBT [13,44]. Although there is limited empirical evidence for the effectiveness of psychotherapy for Indigenous people, culturally responsive approaches are preferred [9,57,72,73]. Strengths-based, culturally adapted, low-intensity CBT and acceptance commitment therapy have been shown to be effective with Aboriginal and Torres Strait Islander populations through randomized controlled trials [16,47].

#### Activity Types

Current dMH tool recommendations suggest that the inclusion of mental health information can prompt early identification and help-seeking [101]. Young people often prefer videos with limited text [62]. Behavioral activation techniques, such as goal-setting, planning, mindfulness, and encouragement of real-world activities, are frequently used strategies in digital health design [44]. Self-monitoring of thoughts, feelings, or behaviors has also demonstrated some evidence of favorable acceptability and effectiveness with adults and young people [44,62,74,102] despite other reports that it is *boring* and *feels like punishment* [103].

#### Engagement

Engagement strategies, such as notifications, social sharing, gamification, and personalization, can improve adherence and intrinsic motivation to dMH apps, if they are delivered and timed appropriately [44,48,62,68,75]. The ability to share information or progress can be particularly salient for cultures with holistic health worldviews; however, it can demotivate some if not aligned with the personal aims of the user [75,76,103]. Notifications should remind users of the benefits and promote opportunities to engage (ie, when feeling stressed or low mood), which can assist in habit formation and motivation [19,35,44,74]. Storytelling through fictional characters aids immersion, models behavior, and allows users to learn skills in a low-risk environment, which can prompt help-seeking [31,77].

#### Interface and Graphic Design

Culturally relevant, intuitive, aesthetic, and minimalist designs increase the acceptability and usability of dMH tools [53,75,78], particularly for those whose first language is not English [79]. Furthermore, mental health apps designed for use by nonclinical populations allow greater accessibility, reduce stigma, and facilitate preventative use [44,61].

#### Language

Language, which is simple, concrete, confident, hopeful, nonclinical, nonbiased, and matched to the literacy level of intended end users, promotes access and adherence to dMH tools [44]. Mechanisms that support and promote literacy, such as audio options, visual prompts, and metaphors, can improve understanding [75,80]. In addition, integration of Indigenous languages can aid communication and engagement for Indigenous people [71,75].

#### Security, Privacy, and Reliability

Apps should include password protection, which can aid the protection of privacy when devices are shared [9,13,81]. Privacy policies should be available, understandable, and regularly updated, with clear reporting of privacy features embedded in the design [13]. Security should also be monitored regularly for breaches.

#### Safety

The Australian National Safety and Quality dMH standards highlight several critical inclusions and considerations for crisis support [13]. The dMH services should include mechanisms for recognizing and responding to acute deterioration in mental state. Links to crisis support should be presented in attractive, easy-to-access ways throughout the app [13].

#### Evidence and Implementation

Although some dMH programs have demonstrated effectiveness with culturally diverse [16,82] and other young people [58,83], overall, there is a paucity of evidence of the effectiveness of mental health apps for adolescents [59,84]. It is also unclear how dMH solutions perform outside trial conditions, with reach, uptake, and adherence remaining problematic, highlighting the need for continued focus on implementation and sustainability [59,85]. Culturally responsive early intervention mental health services are lacking in most regions of Australia, especially in regional and remote areas [73,104]. The dMH interventions can promote access to early intervention, complement existing services, and prompt help-seeking [62,105].

#### Accessibility

Smartphones are the most accessible digital tools for mental health treatment worldwide [44] and are increasingly available to Aboriginal and Torres Strait Islander young people [69,86,87]. However, digital tool design should consider data credit, ongoing data use, offline use, availability free of charge, and platform compatibility [9,13,48].

### Integrating Participant Feedback and the Scientific Literature

#### Overview

Feedback on storyboards and prototypes was sought from a diverse group of participants and sources, generating a range of perspectives and recommendations. Most young people demonstrated unique and strong preferences for specific activity types, storytelling, strengths-based information, character attributes, and gamification features. Service providers showed similar preferences, also highlighting the importance of features to support the psychological approach, accessibility, implementation, and continuity of care. The literature strongly supports CBT approaches, safety and quality features, and the necessity for evidence, which was not disputed by the young people or service providers but was also not prioritized. We reflect on the processes undertaken to engage, integrate multiple views, and prioritize features for inclusion in the following sections.

#### Engagement

One of the key challenges throughout was maintaining young people’s interest and engagement through culturally safe processes over a 2-year time frame. The ongoing engagement of all sites and several young people throughout multiple phases of this project and the rich in-depth data collected suggest that the strategies used were reasonably successful. Strategies to maintain engagement included working within supportive schools and services and employing youth and senior Indigenous researchers with established connections to the community. Continually reviewing and revising co-design activities to meet the needs of the young people and frequently recruiting throughout the data collection process further aided success.

#### Integrating Multiple Views

Another key challenge was the differing youth preferences highlighted among individuals across and within co-design workshop groups. For example, the inclusion of storytelling through characters was a recurring preference. However, differing preferences for the narrative, context, character identities, and depth of information presented were identified. These challenges were compounded by the staggered timing and nonstatic group of participants in co-design workshops. The IYRG engaged a group that was diverse in terms of geographical location, gender, and age. This group was provided the findings from the co-design workshops to ensure they were informed by previous work undertaken. This improved efficiency and led to improved decision-making capacity of young people throughout development.

#### Prioritization

Throughout this participatory design project, we identified app features preferred by participants and assessed their alignment with current recommendations and scientific literature. Although we included many preferred features to some degree, the *available budget and prioritization of best practice recommendations* influenced what was integrated into the prototype. A prioritization process was required, with some features earmarked for later inclusion (Table 3). Prioritization was based on the consideration of young people’s preferences, recommendations from the literature, and study protocols developed for a feasibility study (commenced in August 2020). Selected features, such as notifications and prompted mood monitoring, were popular among young people and recommended in the literature. Given the resource limitations, these were deprioritized in initial development in preference for the diversity of content (ie, variety of strengths, challenges, strategies, and rewards), activities (ie, minigames, videos, and pictorial selection or upload), and appeal (ie, characters of differing age, gender, and geographical location).

**Table 3:** Aboriginal and Islander Mental Health Initiative for Youth (version 1.0) prioritization of app features for development.

App features/Included			Not included owing to resource limitations; considered for next version	Justification
**Therapeutic approach**				
	Low-intensity culturally adapted CBT^a^ intervention (evidence-based)		N/A^b^	G^c^, L^d^, Y^e^, SP^f^
	Stories and prompts to address anxiety and low mood		N/A	L, Y
**Activity types**				
	Videos		N/A	L, Y
	Mental health information with links to further information		N/A	G, L, Y, SP
	Minigames to prompt relaxation and real-world activities		N/A	L, Y
	Self-monitoring of personal goals		Self-monitoring of mood and behavior	L, Y, SP
**Engagement**				
	Storytelling through characters		N/A	L, Y
	Real-time engagement		N/A	L, Y, SP
	Gamification (eg, levels and rewards)		N/A	L, Y
	Progress and summary page		N/A	L, Y
	Ability to complete a personalized quest—add names and photos throughout (customization)		Ability to customize characters and notifications and select voice or language settings	G, L, Y, SP
	N/A		Notifications to remind users of program benefits and encourage use	L, Y
**Interface and design**				
	Simple and intuitive interface		N/A	G, L, Y, SP
	Smooth, easy to use, accurate, and logical flow		N/A	G, L, Y
	Aesthetic and minimalist design		N/A	G, L, Y, SP
	Culturally relevant graphics tailored to target group		N/A	G, L, Y, SP
**Language**				
	Simple, concrete, confident, hopeful, nonclinical, nonbiased language, and matched to the literacy level of users		N/A	G, L, Y, SP
	Mechanisms to support literacy (eg, visual prompts and cues, use of metaphors, and minimal written content)		Mechanisms to support literacy (eg, audio of all app content)	L, Y, SP
	Indigenous language words used within English text in line with stories		Full integration of multiple Indigenous languages throughout	L, Y
**Privacy**				
	Up-to-date, available, and understandable privacy policy		N/A	G, L
	N/A		Regularly monitored security	G, L
	N/A		Password protection—optional	G, L, Y, SP
**Safety**				
	Easily accessible links to crisis support		N/A	G, L, Y, SP
**Evidence and implementation**				
	Experimental trials to examine efficacy		N/A	G, L
	Analytics (use data)		N/A	G, L
	N/A		The ability for users to provide feedback	G, L
	N/A		Assessments embedded with prompts at different time points	G, L
**Accessibility**				
	Compatible with mobile phone—currently only Android		Compatible with all platforms	G, L, Y, SP
	Suitable for supported or self-driven use		N/A	L, SP
	Offline use available		N/A	L, Y
	N/A		Users made aware of data use	G
	N/A		Multi-user tablet version	SP

^a^CBT: cognitive behavioral therapy.

^b^N/A: not applicable.

^c^G: guidelines.

^d^L: recommended in the literature.

^e^Y: youth preference.

^f^SP: service provider preference.

#### App Development

App development occurred through a series of research team meetings with the app developers, involving iterative user testing of developed prototypes. Input from the IYRG was sought by the research team throughout development for important design decisions.

### Outcome: AIMhi-Y App (Version 1.0)

**Figure 5:**
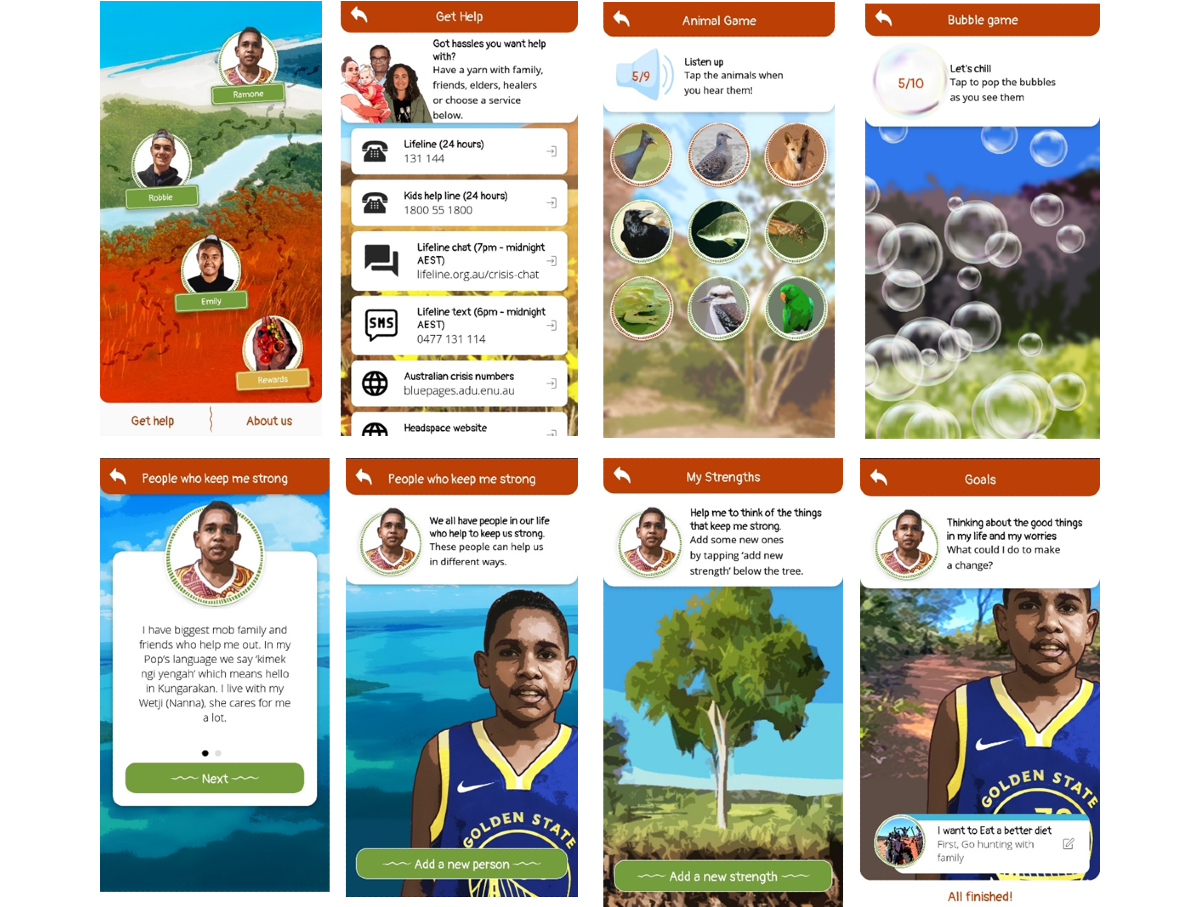
Selected screenshots from Aboriginal and Islander Mental Health Initiative for Youth app (version 1.0).

The product developed is a smartphone-based AIMhi-Y app (version 1.0) that integrates culturally adapted low-intensity CBT, psychoeducation, and mindfulness-based activities into a universal early intervention (Figure 5). The app aims to increase mental health literacy, self-management, and help-seeking for Aboriginal and Torres Strait Islander young people. Users assist fictional characters through a series of quest levels, aiming to become familiar with the content before beginning their own quest. The integrated AIMhi Stay Strong therapy follows a 4-step process: identifying people who keep them strong, strengths, worries, and supported goal-setting, which is interspersed with psychoeducational videos [63]. Activities and information target both anxiety and low mood. A summary page collates user or character information and presents their progress. Minigames promote relaxation; encourage real-world activities; and provide fun, engaging, and immersive sensory experiences. An example is a game in which users listen to an immersive soundtrack and are asked to identify familiar animal sounds by selecting related images. Storytelling aims to develop relationships and facilitate skill development in a safe, nonthreatening environment. The app aims to be easy to use with intuitive designs and nonclinical youth-friendly language. Aboriginal language words relevant to specific characters are integrated throughout. Vibrant colors and design elements reflect the natural landscapes of different Northern Territory (Australia) regions. Help contacts and links for further information are available throughout. A deidentified database captures app use and user interaction with app features.

## Discussion

### Principal Findings

This second phase participatory design project integrated Aboriginal and Torres Strait Islander young people’s and service providers’ preferences with recommendations from the scientific literature to develop the AIMhi-Y app (version 1.0). Young people expressed diverse and strong desires for app features, which were generally well aligned with findings in the scientific literature. Throughout this project, young people have been involved in an iterative design, development, and review process. Several challenges and key learnings have been noted. We reflect on how our processes align with the attributes of successful participatory design.

This project engaged a large, diverse group of young people with varied demographic, cultural, and linguistic backgrounds, many of whom may not usually be involved in co-design processes [38,106]. The planning, reflection, and refinement of culturally responsive co-design activities with the input of 4 Indigenous researchers led to increased engagement and acceptability of our processes. However, maintaining young people’s participation for 2 years during adolescence, where social, emotional, and vocational needs are changing, was difficult: a challenge highlighted by others [106,107]. New participants were recruited throughout the process and they shared new ideas, suggestions, and opinions frequently. Our findings align with other studies that have identified differing preferences based on age, gender, and mental health status [88], suggesting that dMH tools need to be designed and tailored with specific target audiences in mind [33,48,68,108].

The staggered timing of diverse groups across multiple sites and the iterative nature of participatory design challenged our democratic processes: difficulties others have identified [20,36,38]. Each co-design workshop was iteratively modified in response to previous findings, limiting the use of voting or consensus methods [38,37]. Therefore, informed by previous findings, literature, and safety standards, researchers assumed a decision-making role, which shifted power differentials and impacted the extent of young people’s influence in the co-design process. The sustained and regular inclusion of 2 youth and 2 senior Indigenous researchers and an experienced research team optimized youth, clinician, and Aboriginal and Torres Strait Islander influence in decision-making. The inclusion of the IYRG throughout development improved the decision-making capacity of young people in some domains. However, the information and decisions presented to the group were prioritized by the research team to keep within time frame constraints. Systematically taking findings and decisions from one group to the other and back again (eg, through decision logs) might have improved the transparency of our decision-making.

Furthermore, although young people provided many innovative and creative suggestions, budget and timeline restrictions limited what was incorporated into this first prototype app design. This is consistent with the challenges highlighted by others in facilitating a process allowing participants to be creative and innovative while providing rules and guidelines to keep work within the project scope and budget [109]. Finally, determining the degree to which upskilling of participants occurred in a culturally and linguistically diverse, iterative situation, where knowledge from all stakeholders remained both tacit and latent, presented a significant challenge [24]. In addition, the knowledge presented, the responses sought, and the interpretation of feedback by the research team were inevitably influenced by the researchers’ experience, availability, paradigms, and worldviews [110].

Overall, the integration of findings into the app prototype proved complex, requiring consideration of young people’s and service providers’ preferences, recommendations from the literature, and budget. Future iterations will be informed by a feasibility study (commenced in August 2020) and might include additional activities; greater accessibility across platforms and devices; options for customization; self-monitoring; full integration of Aboriginal languages; and additional characters representing diverse ages, genders, language groups, and geographic areas facing differing age-appropriate strengths and challenges. Key learnings throughout this project include the importance of understanding and upskilling all those involved in the co-design project on previous work undertaken, the practical limitations, findings from previous research, and safety recommendations early in the co-design process. Although seeking diverse opinions undoubtedly strengthened our approach, a detailed plan of how to integrate the information across stakeholder groups would have aided our process. Identifying stakeholder roles, opportunities for input, responsibilities, influence, and decision-making strategies across app characteristic domains may have allowed more decision-making opportunities to be presented to young people.

### Limitations

All of the small number of service providers interviewed had existing relationships and interest in the project, likely resulting in favorable comments. However, their involvement ensured the consideration of context and supported seeding ideas for further exploration and testing, thereby strengthening rather than detracting from the process. Furthermore, young people’s engagement in this project was influenced by organizational, cultural, and linguistic contexts; group dynamics; facilitator experience; and availability. The difficulties in engaging an interpreter at the drug rehabilitation site undoubtedly impacted young people’s engagement. The IYRG members were reimbursed for their participation, which may have influenced their comments. However, others have suggested that reimbursement might aid participation by promoting a sense of pride and responsibility [38]. Although sustained engagement facilitated trust and rapport over time, the transient nature of this population group proved challenging as new relationships needed to be established. Finally, the research team took a lead role in decision-making and prioritization of app features owing to resource limitations, impacting the extent of young people’s influence in the co-design process.

### Conclusions

By using a participatory design approach, we engaged a large and diverse group of young people to develop a new culturally informed early intervention mental health treatment app. We identified challenges in reaching consensus, upskilling participants, and allowing equal representation of views in a genuinely participatory process. By describing our processes, we aim to provide transparency in reporting of participatory design approaches. Although the process proved challenging, we successfully created a new app integrating youth preferences and best practice recommendations within a limited time frame and budget. A current feasibility study is underway to evaluate the usability, feasibility, and appropriateness of the AIMhi-Y app and its potential for improving the well-being of Aboriginal and Torres Strait Islander young people.

## Appendix

Multimedia Appendix 1 Service provider interview guide.

Multimedia Appendix 2 Search strategy.

Multimedia Appendix 3 Recommended features for digital mental health tools for Indigenous young people.

## Abbreviations

AIMhi-Y: Aboriginal and Islander Mental Health Initiative for Youth
CBT: cognitive behavioral therapy
dMH: digital mental health
IYRG: Indigenous youth reference group
