# Supplementary material for: Determining Priorities in the Aboriginal and Islander Mental Health Initiative for Youth App Second Phase Participatory Design Project: Qualitative Study and Narrative Literature Review
Source: JMIR Form Res. 2022 Feb 18;6(2):e28342. doi: 10.2196/28342 (PMC8900920; doi:10.2196/28342)
Supplement: Multimedia Appendix 1 [file formative_v6i2e28342_app1.docx]

**Multimedia file one – Service Provider interview schedule**

AIMhi-Y Draft App

Have you had a chance to view the latest version of the AIMhi-Y App Draft?

Do you have any questions?

What do you think of the draft?

Do you have any comments on content, format or usability of the draft app?

Which young people do you perceive might benefit from using this app?

How do you see this app/resource being useful in your school or organization?

How might you/other staff use it with young people?

Which format would it be most helpful to your organization? App – tablet or smartphone, single user, multiuser

Are there any features which you think might prevent your organization taking on this app?
