# Supplementary material for: Determining Priorities in the Aboriginal and Islander Mental Health Initiative for Youth App Second Phase Participatory Design Project: Qualitative Study and Narrative Literature Review
Source: JMIR Form Res. 2022 Feb 18;6(2):e28342. doi: 10.2196/28342 (PMC8900920; doi:10.2196/28342)
Supplement: Multimedia Appendix 2 [file formative_v6i2e28342_app2.docx]

Multimedia file two - Search Strategy

Multiple databases (EBSCOhost, Scopus, CINAHL, PubMed, Google Scholar) and grey literature sites (Google, Informit, Organisation websites) were searched by the first author (JP) between July 2019 and July 2020.

Inclusion criteria

- Articles published in English (due to limitation in resources)
- Articles published from Jan 2000 to July 2020

The following search terms were used in multiple combinations, involving one to four terms linked with ‘AND’:

- (“Mental health” OR psych* OR wellbeing OR “social and emotional wellbeing” OR “substance abuse” OR “substance use” OR drug OR alcohol OR “adolescent wellbeing” OR depression OR stress OR anxiety OR psychosis OR suicide OR distress)
- (“e-mental health” OR technolog* OR “mobile applications” OR computers OR online OR digital)
- (psych* OR therapy OR treatment OR mindfulness OR “motivational interviewing” OR “strengths based” OR “brief intervention” OR therap* OR “cognitive therap*” OR “narrative therap*” OR motivation* OR “strengths based” OR family OR “talking therapy” OR acceptance OR counselling)
- (adolesce* OR youth OR young OR “young adult”)
- (indigenous OR aboriginal OR “Torres strait” OR “first people” OR “first nation people”)
- (trial OR impact OR effectiv* OR improve* OR “randomised controlled trial” OR RCT)
