# Supplementary material for: Determining Priorities in the Aboriginal and Islander Mental Health Initiative for Youth App Second Phase Participatory Design Project: Qualitative Study and Narrative Literature Review
Source: JMIR Form Res. 2022 Feb 18;6(2):e28342. doi: 10.2196/28342 (PMC8900920; doi:10.2196/28342)
Supplement: Multimedia Appendix 3 [file formative_v6i2e28342_app3.docx]

Multimedia appendix three. Recommended features for dMH tools for Indigenous young people

| **Design aspects** | **Recommendation** | **Strength of evidence (major limitation)** |
| --- | --- | --- |
| Therapeutic approach | - Incorporate effective therapeutic approaches such as CBT | One guideline (not population specific) [1]  Three systematic reviews (not Indigenous focus) [2-4]  Four RCT studies (not all youth; not all dMH focus) [5-9]  Two observational studies [10, 11] |
|  | - Culturally adapted or responsive | Five systematic reviews [12-16]  Three systematic reviews (not dMH focus) [17-19]  Two RCT studies (one not dMH focus) [5, 7]  Two qualitative study (not all youth focus) [20, 21]  One expert opinion [22] |
|  | - Address anxiety and low mood | One systematic review [16]  Two systematic reviews (not Indigenous focus) [2, 3] |
|  | - Prevention and early intervention focus | Three systematic reviews (not Indigenous focus) [2, 3, 23] |
| Activity types | - Encourage real-world activities, e.g., exercise, mindfulness, goal setting, and skills training | One framework (not population specific) [24]  Three systematic reviews (non Indigenous focus) [2, 4, 25]  Three RCT’s (not all youth) [5, 7, 26]  One feature article (non-Indigenous focus) [27]  Two qualitative [20, 21, 28] |
|  | - Include mental health information with optional links to in-depth information | Three guidelines (not population specific) [1, 29, 30]  Two systematic reviews (non Indigenous focus) [2, 31]  Descriptive analysis (not Indigenous-specific) [10]  Two qualitative studies [21, 28] |
|  | - Self-monitoring of thoughts, feelings, or behaviours | Four systematic review (non Indigneous focus) [2, 4, 31, 32]  Two qualitative studies [21, 28] |
| Engagement | - Tailoring, personalisation, and customisation | Two guidelines (not population specific) [1, 24]  One systematic review [16]  Six systematic reviews (non Indigneous focus) [2-4, 25, 31-33]  Three qualitative studies [21, 34] |
|  | - Real-time engagement | Three systematic reviews (non Indigenous focus) [2, 31, 35] |
|  | - Gamification, e.g., levels, rewards | One framework (not population specific) [24]  Four systematic reviews (non Indigenous focus) [31, 32, 35]  Two RCT (not all Indigenous focus) [36, 37]  Three qualitative studies [21, 34, 38] |
|  | - Prompts to remind users of the program benefits and encourage use | One guideline (not population specific) [33]  Four systematic reviews (non Indigenous focus) [2, 4, 31, 32]  RCT (not Indigenous focus) [39]  Two qualitative studies [21, 34] |
|  | - Storytelling through characters | One framework (not population specific) [24]  One systematic review (not Indigenous specific) [35]  One RCT (not Indigenous focus) [37]  Four qualitative (one not Indigenous focus) [21, 38, 40, 41] |
| Interface and graphic design | - Accurate performance, flow, ease of use, with a simple and intuitive interface | Four guidelines (not population specific) [1, 24, 29, 30, 42]  Five systematic review (non Indigneous focus) [2, 4, 31, 32, 43]  Three qualitative [20, 21, 44] |
|  | - Culturally relevant, intuitive, aesthetic, and minimalist designs | One guideline (not population specific) [30]  Six systematic reviews (not all Youth) [12, 14-16, 43]  Three systematic reviews (non Indigenous focus) [2, 4, 31]  Mixed methods observational study [44]  Four qualitative studies [20, 28, 34] |
|  | - Freedom of navigation (ever-present home button) | One systematic review (not Indigenous focus) [4]  Mixed method observational study [45] |
| Language | - Simple, confident, hopeful, non-clinical, non-biased language, matched to the literacy level of users | One guideline (not population specific) [1]  Two systematic reviews [16, 43]  Three systematic review (not Indigneous focus) [2, 4, 31]  Three RCT [7, 46, 47]  Two qualitative [48-50] |
|  | - Mechanisms to support literacy, e.g., audio, video, use of metaphors | One systematic review [16]  One systematic review (not Indigenous) [31]  Two RCT [7, 9]  Three qualitative [21, 34, 48] |
|  | - Integration of Indigenous languages | Five qualitative studies [20, 21, 34, 48, 51] |
| Security, privacy, and reliability | - Available and understandable privacy policy | One guideline (not population specific) [1]  One systematic review (non Indigneous focus) [2] |
|  | - System reliability | Three guidelines (not population specific) [1, 29, 30] |
|  | - Regularly monitored security | One guideline (not population specific) [1] |
|  | - Password protection/pin – optional | One guideline (not population specific) [1]  Two qualitative [20, 21] |
| Safety | - Easily accessible links to crisis support and mechanisms to recognise and respond to acute deterioration in mental state | One guideline (not population specific) [1]  One systematic review (non Indigenous focus) [2]  One RCT [7]  Three qualitative studies [20, 21, 52] |
| Evidence and implementation | - Experimental trials to examine efficacy | Three guidelines (not population specific) [1, 30, 33]  Seven systematic reviews (non Indigneous focus) [2-4, 23, 32, 53]  Four RCT’s [6, 26, 37, 54]  Three qualitative (not all Indigneous focus) [21, 55] |
|  | - Assessments embedded with timely prompts | One guideline (not population specific) [1]  One systematic review (non Indigenous focus) [2] |
|  | - Analytics, transparently described to users with the ability for users to provide feedback | One guideline (not population specific) [1]  One systematic review (non Indigenous focus) [2] |
|  | - Collaboration with users and their support people throughout planning, design, evaluation, and implementation | One guideline (not population specific) [1]  Five systematic reviews [12, 14-16, 43]  Two systematic review (non Indigneous focus) [2, 25] |
|  | - Focus on implementation and sustainability | Four systematic reviews (non Indigenous focus) [2, 3, 31, 53, 56] |
| Accessibility | - Mobile phone compatible | Four systematic reviews (non Indigneous focus) [2, 3, 25, 32, 55, 57]  Four observational studies [46, 58-60]  Six qualitative studies [21, 44, 61, 62] |
|  | - Supported use | One systematic review [16]  Two systematic reviews (not Indigenous focus) [31, 63]  One qualitative study [20, 55] |
|  | - Data credit, ongoing data usage, offline use, availability free of charge, and platform compatibility considerations | One guideline (not population specific) [1]  Two systematic reviews [14, 16]  Two qualitative studies [20, 21] |

References:

1. Australian Commission on Safety and Quality in Health Care. National Safety and Quality Digital Mental Health Standards. Canberra: Australian Commission on Safety and Quality in Health Care,, 2020.

2. Bakker D, Kazantzis N, Rickwood D, Rickard N. Mental Health Smartphone Apps: Review and Evidence-Based Recommendations for Future Developments. JMIR mental health. 2016;3(1):e7-e. PMID: 26932350. doi: 10.2196/mental.4984.

3. Clarke AM, Kuosmanen T, Barry MM. A Systematic Review of Online Youth Mental Health Promotion and Prevention Interventions. Journal of Youth and Adolescence. 2015;44(1):90-113. doi: 10.1007/s10964-014-0165-0.

4. Huguet A, Rao S, McGrath PJ, Wozney L, Wheaton M, Conrod J, et al. A Systematic Review of Cognitive Behavioral Therapy and Behavioral Activation Apps for Depression. PLoS One. 2016;11(5):e0154248. doi: 10.1371/journal.pone.0154248.

5. Nagel T, Robinson G, Condon J, Trauer T. Approach to treatment of mental illness and substance dependence in remote Indigenous communities: results of a mixed methods study. The Australian journal of rural health. 2009 Aug;17(4):174-82. PMID: 19664081. doi: 10.1111/j.1440-1584.2009.01060.x.

6. Fleming T, Dixon R, Frampton C, Merry S. A pragmatic randomized controlled trial of computerized CBT (SPARX) for symptoms of depression among adolescents excluded from mainstream education. Behavioural and cognitive psychotherapy. 2012;40(5):529-41. PMID: 22137185. doi: 10.1017/S1352465811000695.

7. Tighe J, Shand F, Ridani R, Mackinnon A, De La Mata N, Christensen H. Ibobbly mobile health intervention for suicide prevention in Australian Indigenous youth: a pilot randomised controlled trial. BMJ open. 2017;7(1):e013518. PMID: 28132007. doi: 10.1136/bmjopen-2016-013518.

8. Dingwall KM, Nagel T, Hughes JT, Kavanagh DJ, Cass A, Howard K, et al. Wellbeing intervention for chronic kidney disease (WICKD): a randomised controlled trial study protocol. BMC psychology. 2019;7(1):2. PMID: 30621791. doi: 10.1186/s40359-018-0264-x.

9. Dingwall KM, Sweet M, Cass A, Hughes JT, Kavanagh D, Howard K, et al. Effectiveness of Wellbeing Intervention for Chronic Kidney Disease (WICKD): results of a randomised controlled trial. BMC nephrology. 2021;22(1):136. PMID: 33866968. doi: 10.1186/s12882-021-02344-8.

10. Titov N, Dear B, Nielssen O, Staples L, Hadjistavropoulos H, Nugent M, et al. ICBT in routine care: A descriptive analysis of successful clinics in five countries. Internet Interventions. 2018 2018/09/01/;13:108-15. doi: https://doi.org/10.1016/j.invent.2018.07.006.

11. Titov N, Schofield C, Staples L, Dear BF, Nielssen O. A comparison of Indigenous and non-Indigenous users of MindSpot: an Australian digital mental health service. Australasian psychiatry : bulletin of Royal Australian and New Zealand College of Psychiatrists. 2019;27(4):352-7. PMID: 30058351. doi: 10.1177/1039856218789784.

12. Hensel JM, Ellard K, Koltek M, Wilson G, Sareen J. Digital Health Solutions for Indigenous Mental Well-Being. Current psychiatry reports. 2019;21(8):68. PMID: 31263971. doi: 10.1007/s11920-019-1056-6.

13. Brusse C, Gardner K, McAullay D, Dowden M. Social Media and Mobile Apps for Health Promotion in Australian Indigenous Populations: Scoping Review. J Med Internet Res. 2014;16(12):e280. PMID: 25498835. doi: 10.2196/jmir.3614.

14. Jones L, Jacklin K, O'Connell ME. Development and Use of Health-Related Technologies in Indigenous Communities: Critical Review. J Med Internet Res. 2017;19(7):e256. PMID: 28729237. doi: 10.2196/jmir.7520.

15. Hobson GR, Caffery LJ, Neuhaus M, Langbecker DH. Mobile Health for First Nations Populations: Systematic Review. JMIR Mhealth Uhealth. 2019;7(10):e14877. PMID: 31593537. doi: 10.2196/14877.

16. Toombs E, Kowatch KR, Dalicandro L, McConkey S, Hopkins C, Mushquash CJ. A systematic review of electronic mental health interventions for Indigenous youth: Results and recommendations. Journal of telemedicine and telecare. 2020:1357633X19899231. PMID: 31937199. doi: 10.1177/1357633X19899231.

17. Pomerville A, Burrage RL, Gone JP. Empirical Findings From Psychotherapy Research With Indigenous Populations: A Systematic Review. Journal of consulting and clinical psychology. 2016.

18. Antonio M, Chung-Do J. Systematic review of interventions focusing on Indigenous adolescent mental health and substance use. American Indian and Alaska Native Mental Health Research (Online). 2015;22(3):36-56. doi: 10.5820/aian.2203.2015.36.

19. MacLean S, Ritte R, Thorpe A, Ewen S, Arabena K. Health and wellbeing outcomes of programs for Indigenous Australians that include strategies to enable the expression of cultural identities: a systematic review. Aust J Prim Health. 2017 Sep;23(4):309-18. PMID: 28619126. doi: 10.1071/py16061.

20. Povey J, Mills PPJR, Dingwall KM, Lowell A, Singer J, Rotumah D, et al. Acceptability of Mental Health Apps for Aboriginal and Torres Strait Islander Australians: A Qualitative Study. Journal of medical Internet research. 2016;18(3):e65. PMID: 26969043. doi: 10.2196/jmir.5314.

21. Povey J, Sweet M, Nagel T, Mills PPJR, Stassi CP, Puruntatameri AMA, et al. Drafting the Aboriginal and Islander Mental Health Initiative for Youth (AIMhi-Y) App: Results of a formative mixed methods study. Internet Interventions. 2020;21. doi: 10.1016/j.invent.2020.100318.

22. Westerman T. Engaging Australian Aboriginal youth in mental health services. Australian Psychologist. 2010;45(3):212-22. doi: 10.1080/00050060903451790.

23. Richardson T, Stallard P, Velleman S. Computerised Cognitive Behavioural Therapy for the Prevention and Treatment of Depression and Anxiety in Children and Adolescents: A Systematic Review. Clinical Child and Family Psychology Review. 2010;13(3):275-90. doi: 10.1007/s10567-010-0069-9.

24. Cheek C, Fleming T, Lucassen MF, Bridgman H, Stasiak K, Shepherd M, et al. Integrating Health Behavior Theory and Design Elements in Serious Games. JMIR Ment Health. 2015 Apr-Jun;2(2):e11. PMID: 26543916. doi: 10.2196/mental.4133.

25. Orji R, Moffatt K. Persuasive technology for health and wellness: State-of-the-art and emerging trends. Health Informatics Journal. 2018;24(1):66-91. doi: 10.1177/1460458216650979.

26. Dingwall KM. Wellbeing Intervention for Chronic Kidney Disease: A Randomised Controlled Trail In: Povey J, editor. Email communication ed2018.

27. Kappen D, Orji R. Gamified and persuasive systems as behavior change agents for health and wellness. XRDS: Crossroads, The ACM Magazine for Students. 2017;24(1):52-5. doi: 10.1145/3123750.

28. Black Dog Institute. The extension of iBobbly: an app to reduce suicidality among young Aboriginal and Torres Strait Islander people. New South Wales, Australia: Black Dog Institute, 2015.

29. Stoyanov S, Hides L, Kavanagh D, Wilson H. Development and Validation of the User Version of the Mobile Application Rating Scale (uMARS). JMIR Mhealth Uhealth. 2016 Jun 10;4(2):e72. PMID: 27287964. doi: 10.2196/mhealth.5849.

30. Stoyanov S, Hides L, Kavanagh D, Zelenko O, Tjondronegoro D, Mani M. Mobile app rating scale: a new tool for assessing the quality of health mobile apps. JMIR mHealth and uHealth. 2015;3(1):e27-e. PMID: 25760773. doi: 10.2196/mhealth.3422.

31. Liverpool S, Mota CP, Sales CMD, Čuš A, Carletto S, Hancheva C, et al. Engaging Children and Young People in Digital Mental Health Interventions: Systematic Review of Modes of Delivery, Facilitators, and Barriers. J Med Internet Res. 2020;22(6):e16317. PMID: 32442160. doi: 10.2196/16317.

32. Perski O, Blandford A, West R, Michie S. Conceptualising engagement with digital behaviour change interventions: a systematic review using principles from critical interpretive synthesis. Translational behavioral medicine. 2017 2017/06//;7(2):254-67. PMID: 27966189. doi: 10.1007/s13142-016-0453-1.

33. Orji R, Mandryk RL. Developing culturally relevant design guidelines for encouraging healthy eating behavior. International Journal of Human - Computer Studies. 2014;72(2):207. doi: 10.1016/j.ijhcs.2013.08.012.

34. Shepherd M, Merry S, Lambie I, Thompson A. Indigenous Adolescents’ Perception of an eMental Health Program (SPARX): Exploratory Qualitative Assessment. JMIR Serious Games. 2018;6(3):e13. PMID: 29980495. doi: 10.2196/games.8752.

35. Johnson D, Deterding S, Kuhn K-A, Staneva A, Stoyanov S, Hides L. Gamification for health and wellbeing: A systematic review of the literature. Internet Interventions. 2016 2016/11/01/;6:89-106. doi: https://doi.org/10.1016/j.invent.2016.10.002.

36. Kelders SM, Sommers-Spijkerman M, Goldberg J. Investigating the Direct Impact of a Gamified Versus Nongamified Well-Being Intervention: An Exploratory Experiment. J Med Internet Res. 2018;20(7):e247. PMID: 30049669. doi: 10.2196/jmir.9923.

37. Merry S, Stasiak K, Shepherd M, Frampton C, Fleming T, Lucassen M. The effectiveness of SPARX, a computerised self help intervention for adolescents seeking help for depression: Randomised controlled non-inferiority trial. British Medical Journal. 2012 2012-04-19 22:32:09;344. doi: 10.1136/bmj.e2598.

38. Christie GI, Shepherd M, Merry SN, Hopkins S, Knightly S, Stasiak K. Gamifying CBT to deliver emotional health treatment to young people on smartphones. Internet Interventions. 2019 2019/10/18/:100286. doi: https://doi.org/10.1016/j.invent.2019.100286.

39. Titov N, Dear BF, Johnston L, McEvoy PM, Wootton B, Terides MD, et al. Improving adherence and clinical outcomes in self-guided internet treatment for anxiety and depression: A 12-month follow-up of a randomised controlled trial. PLoS ONE. 2014;9(2):1-8. PMID: 94731191. doi: 10.1371/journal.pone.0089591.

40. Heilemann MV, Martinez A, Soderlund PD. A Mental Health Storytelling Intervention Using Transmedia to Engage Latinas: Grounded Theory Analysis of Participants’ Perceptions of the Story’s Main Character. J Med Internet Res. 2018;20(5):e10028. PMID: 29720357. doi: 10.2196/10028.

41. Nagel T, Thompson C. AIMHI NT 'mental health story teller mob': Developing stories in mental health. Australian e-Journal for the Advancement of Mental Health. 2007;6(2):1-6.

42. Hides L, Kavanagh D, Stoyanov SR, Zelenko O, Tjondronegoro D, Mani M. Mobile Application Rating Scale (MARS): A new tool for assessing the quality of health mobile applications. Melbourne: Young and Well Cooporative Research Centre 2014.

43. Reilly R, Stephens J, Micklem J, Tufanaru C, Harfield S, Fisher I, et al. Use and uptake of web-based therapeutic interventions amongst Indigenous populations in Australia, New Zealand, the United States of America and Canada: a scoping review. Systematic Reviews. 2020 2020/05/31;9(1):123. doi: 10.1186/s13643-020-01374-x.

44. Kral I. Plugged in: Remote Australian Indigenous youth and digital culture. Canberrra: Australian National University, 2010.

45. Orji R, Reilly D, Oyibo K, Orji FA. Deconstructing persuasiveness of strategies in behaviour change systems using the ARCS model of motivation. Behaviour & Information Technology. 2019;38(4):319-35. doi: 10.1080/0144929X.2018.1520302.

46. Tighe J, Shand F, McKay K, Mcalister T-J, Mackinnon A, Christensen H. Usage and Acceptability of the iBobbly App: Pilot Trial for Suicide Prevention in Aboriginal and Torres Strait Islander Youth. JMIR Ment Health. 2020;7(12):e14296. PMID: 33258782. doi: 10.2196/14296.

47. Shand F, Mackinnon A, O’Moore K, Ridani R, Reda B, Hoy M, et al. The iBobbly Aboriginal and Torres Strait Islander app project: Study protocol for a randomised controlled trial. Trials. 2019 2019/04/05;20(1):198. doi: 10.1186/s13063-019-3262-2.

48. Nagel T, Sweet M, Dingwall KM, Puszka S, Hughes JT, Kavanagh DJ, et al. Adapting wellbeing research tools for Aboriginal and Torres Strait Islander people with chronic kidney disease. BMC nephrology. 2020;21(1):130. PMID: 32293331. doi: 10.1186/s12882-020-01776-y.

49. Cass A, Lowell A, Christie M, Snelling P, Flack M, Marrnganyin B, et al. Sharing the true stories: Improving communication between Aboriginal patients and healthcare workers. Medical Journal of Australia. 2002;176(10):466.

50. Lowell A, Maypilama E, Yikaniwuy S, Rrapa E, Williams R, Dunn S. "Hiding the story": indigenous consumer concerns about communication related to chronic disease in one remote region of Australia. International journal of speech-language pathology. 2012 Jun;14(3):200-8. PMID: 22443611. doi: 10.3109/17549507.2012.663791.

51. Davies J, Bukulatjpi S, Sharma S, Caldwell L, Johnston V, Davis JS. Development of a Culturally Appropriate Bilingual Electronic App About Hepatitis B for Indigenous Australians: Towards Shared Understandings. JMIR research protocols. 2015 Jun 10;4(2):e70. PMID: 26063474. doi: 10.2196/resprot.4216.

52. Larsen ME, Nicholas J, Christensen H. A Systematic Assessment of Smartphone Tools for Suicide Prevention. PLoS One 2016;11(4):e0152285. doi: 10.1371/journal.pone.0152285.

53. Grist R, Porter J, Stallard P. Mental Health Mobile Apps for Preadolescents and Adolescents: A Systematic Review. J Med Internet Res. 2017;19(5):e176. PMID: 28546138. doi: 10.2196/jmir.7332.

54. Tighe J, Shand F, Ridani R, Mackinnon A, De La Mata N, Christensen H. Ibobbly mobile health intervention for suicide prevention in Australian Indigenous youth: a pilot randomised controlled trial. BMJ Open. 2017;7(1). doi: 10.1136/bmjopen-2016-013518.

55. March S, Donovan CL, Baldwin S, Ford M, Spence SH. Using stepped-care approaches within internet-based interventions for youth anxiety: Three case studies. Internet Interventions. 2019 2019/09/10/:100281. doi: https://doi.org/10.1016/j.invent.2019.100281.

56. Fleming T, Bavin L, Lucassen M, Stasiak K, Hopkins S, Merry S. Beyond the Trial: Systematic Review of Real-World Uptake and Engagement With Digital Self-Help Interventions for Depression, Low Mood, or Anxiety. J Med Internet Res. 2018;20(6):e199. doi: 10.2196/jmir.9275.

57. Fleming T, Merry S, Stasiak K, Hopkins S, Patolo T, Ruru S, et al. The Importance of User Segmentation for Designing Digital Therapy for Adolescent Mental Health: Findings From Scoping Processes. JMIR Ment Health. 2019;6(5):e12656. PMID: 31066705. doi: 10.2196/12656.

58. McNair Innovations. Indigneous Media and Communications. 2016 [27/12/2019]; Available from: https://mcnair.com.au/wp-content/uploads/Indigenous-Media-Infographic.pdf.

59. McNair Innovations Research. Media useage amongst Aboriginal and Torres Strait people. 2014.

60. Taylor A. Information communication technologies and new Indigenous mobilities? Insights from remote Northern Territory Communities. Journal of rural and Community Development. 2012;7(1):59-73.

61. Kral I. Youth media as cultural practice: remote indigenous youth speaking out loud. Aust Aborig Stud. 2011 2011//;1.

62. Kral I. Shifting perceptions, shifting identities: communication technologies and the altered social, cultural and linguistic ecology in a remote indigenous context. Aus J Anthropol. 2014 2014//;25. doi: 10.1111/taja.12087.

63. Ballegooijen Wv, Cuijpers P, Straten Av, Karyotaki E, Andersson G, Smit JH, et al. Adherence to Internet-Based and Face-to-Face Cognitive Behavioural Therapy for Depression: A Meta-Analysis: e100674. PLoS One, 2014;9(7). doi: 10.1371/journal.pone.0100674.
